# Do bumblebees have signatures? Demonstrating the existence of a speed-curvature power law in *Bombus terrestris* locomotion patterns

**DOI:** 10.1371/journal.pone.0226393

**Published:** 2020-01-15

**Authors:** Laura James, T. G. Emyr Davies, Ka S. Lim, Andrew Reynolds

**Affiliations:** Rothamsted Research, West Common, Harpenden, Hertfordshire, England, United Kingdom; Bruno Kessler Foundation, ITALY

## Abstract

We report the discovery that *Bombus terrestris audax* (Buff-tailed bumblebee) locomotor trajectories adhere to a speed-curvature power law relationship which has previously been found in humans, non-human primates and *Drosophila* larval trajectories. No previous study has reported such a finding in adult insect locomotion. We used behavioural tracking to study walking *Bombus terrestris* in an arena under different training environments. Trajectories analysed from this tracking show the speed-curvature power law holds robustly at the population level, displaying an exponent close to two-thirds. This exponent corroborates previous findings in human movement patterns, but differs from the three-quarter exponent reported for *Drosophila* larval locomotion. There are conflicting hypotheses for the principal origin of these speed-curvature laws, ranging from the role of central planning to kinematic and muscular skeletal constraints. Our findings substantiate the latter idea that dynamic power-law effects are robust, differing only through kinematic constraints due to locomotive method. Our research supports the notion that these laws are present in a greater range of species than previously thought, even in the bumblebee. Such power laws may provide optimal behavioural templates for organisms, delivering a potential analytical tool to study deviations from this template. Our results suggest that curvature and angular speed are constrained geometrically, and independently of the muscles and nerves of the performing body.

## 1. Introduction

At any point along a curve there is a unique circle or line which most closely approximates the curve near that location. The radius of that circle defines the ‘radius of curvature’, R, whilst curvature, C, is defined to be its reciprocal, 1/R. According to this definition, it can be expected that straight lines will have zero curvature, and for a given observer at a fixed scale large circles will have small curvature and small circles will have high curvature. Curvature along with angular speed, A, has been used to quantify human writing signatures[1].

Remarkably the human signature, a powerful individual identifier, adheres to a speed-curvature power law[1]. The speed-curvature, or two-thirds, power law dictates that the instantaneous angular speed of movements vary proportionally to two-thirds power of their curvature[1]. According to the law, movements under high curvature tend to slow down, whereas movements under low curvature speed up[2]. The law is given by

$$A={kC}^{2/3}$$ (1)

where k is a constant of proportionality.

Maximally-smooth movements, which minimize rates of change of acceleration (i.e., jerks and jolts), are generated under the two-thirds power law[3–5], which holds true across a range of voluntary human movements, including drawing, walking and pursuit eye movements[1,3,6,7]. The law also holds true across a diverse range of taxa. The law has been observed in the motor cortical control of Rhesus monkey hand movements whilst drawing [8], and even in the larval movement of the fruit fly (*Drosophila melanogaster*)[5] albeit with a marginally different power-law exponent, three quarters rather than two thirds.

The principal origins of this speed-curvature power law are contentious. One hypothesis suggests that the law results from central planning constraints imposed by the nervous system[8,9]. Another, that the law arises due to physiological constraints conferred by muscular properties and kinematics[2,5,10]. A further view, that the law exists to maximize movement smoothness and minimize jerk[3,9]. Identifying the generative mechanism holds the key to understanding the statistical law, the occurrence of which is remarkable given that behaviours are shaped by individual psyches and by complex social and environmental interactions. It’s identification may help to elucidate how other statistical regularities can occur within the complex movement patterns that arise in nature[11–16]. Progress towards identifying the underlying mechanism can be made by determining the pervasiveness of the two-thirds law, and by establishing whether or not it occurs in other modes of locomotion.

Given that the locomotive patterns of *Bombus terrestris*, and indeed animal organisms, are probably shaped by their motivational states and by environmental factors, a seemingly natural null hypothesis would be that individuals have unique locomotive patterns and that statistical regularities are absent or trivial (for example, a tendency to move forwards with near constant speed). Therefore, to determine the pervasiveness of the law, we must first determine whether the speed-curvature power law persists in the walking trajectories of the bumblebee at all and, if it does, whether the law differs depending on a bee’s environment. We must then determine whether the exponent of the law adheres closely to the two thirds exponent. Finally, it is necessary to also assess whether the power law is the best mathematical descriptor of walking bumblebee trajectories or whether an alternative better describes the relationship.

Walking is distinctly different from the crawling movements made by limbless larvae[17]. Therefore, we might predict that walking bee trajectories would adhere more closely to the two-thirds power law exponent reported for unconstrained movements such as human drawing and walking[1,6], than the three-quarters exponent reported for the mechanically constrained movements of larvae[5].

To the best of our knowledge the speed-curvature power law has not been studied in any other invertebrate other than *Drosophila melanogaster* larvae[5] and never in the final, adult stage of an insect. Here, we report that *Bombus terrestris audax*, a social bumblebee species with a complex behavioural repertoire, displays a two-thirds speed-curvature power law whilst walking in an arena, under differing environments.

## 2. Methods

### Bee subjects

All subjects were *Bombus terrestris audax* from research hives obtained from Biobest Belgium NV (Westerlo, Belgium). Colonies were settled in wooden nest boxes (29 x 21 x 16 cm) and provided with biogluc (Biobest Belgium NV, Westerlo, Belgium) in two gravity feeders in a Perspex foraging tunnel (26 ×4×4 cm) connected to the nest box. Pollen was also provided in baskets in the Perspex tunnel. Gravity feeders and pollen were replenished, as necessary, to ensure a consistent supply of food to the colony. Newly emerged individuals were marked in colour groups by age cohort with coloured plastic bee marking tags (EH Thorne Ltd, Market Rasen, UK) superglued to the top of the thorax. This allows tracking of an individual’s age. All individuals used in a single trial were one-week post-emergence (to allow bees to begin foraging and to be monitored) and of the same age cohort. The hive was observed each day and foragers of each age cohort were identified in the foraging tube by their colour and number. From the foragers recorded in each age cohort ten individuals were randomly selected to be tested per trial. The selected individuals are then randomly allocated to either the treatment or control groups for each trial. Trials were replicated 3 times; all treatments replicated 3 times across 3 different hives.

### The experimental arena

**Fig 1:**
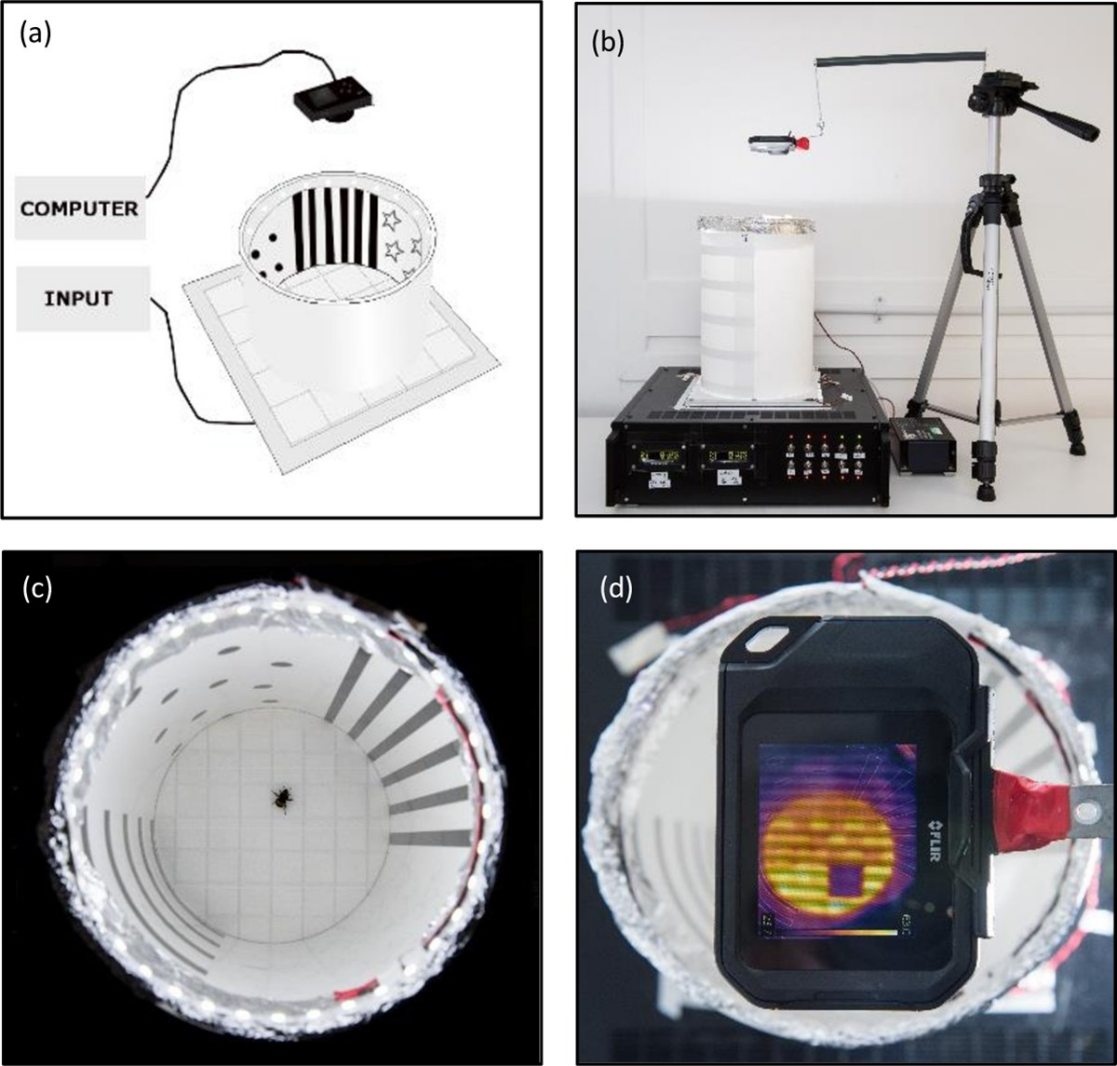
The thermal-visual arena. (a) Diagrammatic representation of the thermal-visual arena. (b) The arena *in-situ* in the lab. (c) A birds-eye view of the arena with an example *Bombus terrestris* forager completing a training trial. (d) A thermal camera being used pre-training trial to confirm the location of the inconspicuous cool reward zone within the arena.

Experiments were conducted within a thermal-visual arena (Fig 1A–1D), similar to a platform previously used for Drosophila tracking[18]. The arena enables the creation of controlled, but naturalistic, environments. A Peltier array of 64 2.5x2.5 cm individually controllable thermoelectric Peltier elements, arranged in an 8x8 grid, facilitates control of the arena’s floor temperature. The arena’s floor is covered in white masking tape to create an inconspicuous, featureless surface which can be easily cleaned and replaced between trials to prevent the use of scent marks by foragers to locate arena rewards. In the training trials, visual patterns were adhered to the surface of the arena’s walls to create a visual landscape consisting of repeating patterns of stars, dots, horizontal and vertical bars, denoting the four quadrants of the arena’s circumference. Light-emitting diodes (LEDs) (colour temperature 6500K) around the top edge of the arena were used to light the arena consistently above the bee flicker fusion frequency [19] (Fig 1C). The arena was kept in a controlled environment room at 22^0^ C with a day: night cycle of 16:8 hr.

### Training environments

The task required forager bees to use visual landscape patterns to locate a reward zone within the arena, in response to four training environments: 1) control environment with no reward or punishment, 2) appetitive reward environment (0.1ml 50% sucrose solution in reward zone), 3) aversive punishment environment (heated arena floor (45°C), cool (25°C) reward zone) and 4) combined aversive and appetitive environment (heated arena floor (45°C), 0.1ml 50% sucrose solution in cool (25°C) reward zone). All rewards (cool zone or sucrose) were inconspicuous and not visually distinguishable from any other tiles on the arena floor.

### Training regime

None of the test subjects had experience of the thermal-visual arena prior to the training trials. Each bee was given ten trials in the arena (each trial was of three minutes duration) spaced across three days. Spaced conditioning, in which temporal spacing exists between successive conditioning trials, has been shown to lead to higher memory consolidation in bees, especially at long intervals [20]. When placed into the thermal visual arena, bees were confined under a clear plastic tube for one minute prior to the trial start, to allow orientation within the arena. The tube was then removed, and the three-minute trial started. All bees were starved for one hour prior to trial start to motivate individuals in the appetitive condition and to remove starvation as a confounding variable between treatments. Bees were confined to individual cages in-between trials to prevent further foraging experience not in the arena and standardise the amount of foraging experience in the arena each bee received. Cages were placed next to each other and adjacent to the hive to allow visual and olfactory communication between hive members.

### Trajectory tracking

To facilitate 2D trajectory tracking, foragers were confined to walking on the test platform by wing clipping. Selected foragers’ wings were clipped using a queen marking cage and dissection scissors (EH Thorne Ltd, Market Rasen, UK).

Individual bee trajectories were filmed using a camera (FLIR C2 Infrared Camera) attached to a tripod above the arena (Fig 1B). Video recording was at four frames per second for ten, three-minute trials per bee. Video files were tracked using CTRAX: the Caltech Multiple Walking Fly Tracker software[21]. The raw centroid tracking data files outputted by CTRAX were then used for speed-curvature power law calculation.

### Speed-curvature power law calculation

For the data analysis, the x, y coordinates and corresponding timestamps for whole trajectories, for individual bees, from the centroid tracking were used to compute angular speed A(t) and curvature C(t) using standard differential geometry[22]. Velocities were calculated from consecutive, regularly timed, positional fixes, $\dot{x}=\frac{x\left(t+\Delta t\right)-x\left(t\right)}{\Delta t}$ and $\dot{y}=\frac{y\left(t+\Delta t\right)-y\left(t\right)}{\Delta t}$ where Δ*t* = 0.2 *s* is the time interval between consecutive recordings. Accelerations $\ddot{x}$ and $\ddot{y}$ were calculated in a directly analogous way from consecutive velocities. Together these quantities determine the ‘radius of curvature’^36^,

$$R=\left|\frac{{\left({\dot{x}}^{2}+{\dot{y}}^{2}\right)}^{3/2}}{\dot{x}\ddot{y}-\dot{y}\ddot{x}}\right|$$ (2)

which in turn gives the angular speed,

$$A={\left({\dot{x}}^{2}+{\dot{y}}^{2}\right)}^{1/2}/R$$ (3)

and the curvature,

C=1/R (4)

### Data selection

Whole trajectories were analysed, with data selected so that only individual bee tracks which had greater than 50 data points (n = >50) were used for analyses (for all other tracks n = between 66 and 1047). Excluded bees: n = 14. Bees used for analysis, n = 45. When we removed all bees with under 100 data points the outcomes of our analyses did not change and therefore we can consider selection at 50 data points to be robust and there was no need to exclude further bees. Data were not filtered (smoothed) prior to processing. Filtering does not affect the outcomes of our analyses (see S1 Appendix).

### Statistical analysis

The hallmark of a power-law relationship between curvature, C, and angular speed, A, is a straight-line relationship between log(C) and log (A). Taking the logarithm of both sides of the two-thirds power-law rule gives the linear relationship log A = log K + beta log C, with β = 2/3. Here, following Zago et al.[5] we looked for such relationships by least squares linear regression of log(C) and log(A). Using this method, we estimated the exponent, β, and the variance, r^2^, accounted for by the power-law.

The power-law scaling demonstrated by our analysis extends over two or more scales of magnitude. This fulfils Stumpf and Porter’s[23] ‘rule of thumb’; after critically appraising power laws identified in biological systems, they suggested that a candidate power law probability frequency distribution should apply over at least two orders of magnitude along both axes and should be explainable by a viable mechanism.

We then went beyond previous analyses[5,24] by comparing our observations with strongly competing functions that resemble power-laws but are not underpinned mechanistically. The power-law relationship between curvature and angular speed cannot, of course, extend to arbitrarily large curvatures and angular speeds because of physiological constraints that place limits on the tightness of turning and on the speed that can be attained by an individual. Departures from power-law are expected when the maximum curvatures and speeds are approached by an individual. Here we examine this by fitting our data to two functions that resemble power-laws over a range of scales, but which depart from power-laws when curvatures and speeds are sufficiently high. These functions are stretched exponentials (which include exponentials as a special case),

$$A=a.\exp\left(bC^{p}\right)$$

and log-normal like functions,

$$A=a.\exp\left(b{\left(\ln C-\ln d\right)}^{2}\right)$$

where a, b, p and d are free parameters that are determined by fitting the functions to our data. The relative merits of the power-law, stretched exponential and log-normal functions as representations of our data were determined using the Akaike information criterion[25].

The stretched exponential and the log-normal like functions can be considered as strongly competing descriptions of our data that contain three rather than two free parameters. This extra flexibility could result in better fits to our data. Functions were fitted to individuals’ movement patterns, rather than to pooled data as we sought to capture an individual’s constraints. We then compared the pooled data with functions parameterized in terms of the average best fit parameters.

Stretched exponentials (typically with p~0.007) provided good fits to our data, but better fits are obtained with power-laws. Even better fits were obtained with the log-normal like functions which is not surprising given that they are more flexible than simple power-laws. In all cases, the Akaike weights for the log-normal like functions are 1.00 which indicates that the log-normal like functions are convincingly favoured over the power-law and stretched exponential functions. However, as is often the case, the better fit of the complex model (the log-normal like function) trades off with the elegance and clarity of the simpler model (the power-law function). The log-normal functions are, however, convex with maxima at lnC = lnd. Such maxima are not evident in our observations and consequently the estimates for lnd (approximately 35) were much larger than lnC_max_ (approximately ten). This implies that the fitted log-normal like functions are effectively fits to power-laws because when lnd are much larger than lnC_max_

$$\begin{array}{l} A\approx a.\exp\left(-2b\ln\left(d\right)\ln(C)+b\ln{\left(d\right)}^{2}\right)\\ \;=kC^{\beta } \end{array}$$

where *k* = *a*.exp(*b*ln(*d*)^2^) and *β* = −2*b*ln(*d*).

Our mean estimates for the power-law exponents; 0.59 (controls, n = 14, range 0.42–0.87), 0.61 (appetitive + aversive, n = 12, range 0.43–0.87), 0.60 (aversive, n = 7, range 0.49–0.94) and 0.57 (appetitive, n = 12, range 0.44–0.8) are broadly consistent with the two-thirds power-law rule. We have therefore arrived at this law using two different approaches; by fitting our data to power-laws and by fitting our data to log-normal functions.

**Fig 2:**
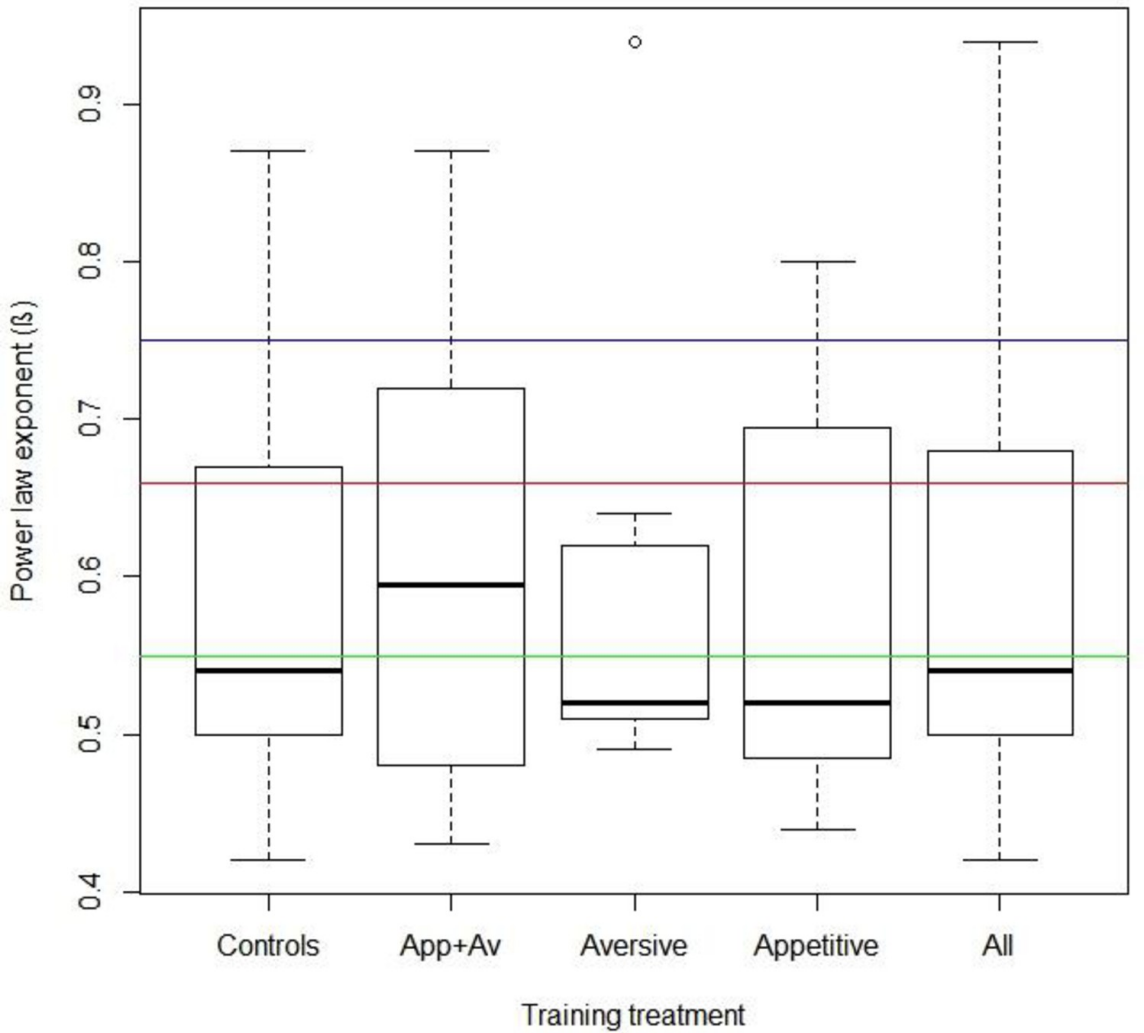
Summary boxplot statistics for the β-exponent of bees in the four conditions: control (n = 14), aversive (n = 7), appetitive (n = 12) and aversive + appetitive (n = 12 (post data filtering) and individuals from all conditions combined. 99% of all data lies within the boxplot whiskers (outliers represented as dots). The two-thirds power exponent (0.66) is represented by the red line. The three-quarters exponent (0.75) by the blue line and a new predicted exponent of 0.55 by the green line. Although treatment groups did not differ significantly from the two thirds exponent (Kruskal-Wallis analysis), when visualised, it is clear that median β-exponent values vary around a 0.55 power exponent value, suggesting that an exponent range of 0.5 to 0.66 best describes the exponents of our walking bees.

Statistically significant differences between the power exponents (β) of treatment groups and expected exponent values of two thirds (0.66) and three quarters (0.75) were calculated using non-parametric tests (Kruskal-Wallis ANOVA by ranks), as data were not normally distributed (Shapiro-Wilk test, p value = 0.000587518***). Kruskal-Wallis tests were conducted in RStudio (Version 1.0.44–2009–2016 RStudio, Inc.). Summary boxplot, Fig 2 was produced in RStudio using the ‘ggplot’ package.

## 3. Results

### Varying exploratory strategies

To facilitate the creation of different walking trajectories, bees were tested across differing training environments within a thermal-visual arena (Fig 1). Training environments differed in the reward or incentive provided to foragers, providing either no reward or punishment (control), an appetitive sucrose reward, an aversive punishment (heated arena floor) or a combined aversive punishment and appetitive reward environment. Each bee was given ten training trials, experiencing only one of the training environments across all ten trials. In each training trial bees were required to use visual landscape patterns, around the circumference of the arena, to locate the appropriate reward zone (refer to ‘training environments’ in methods section for further details).

**Fig 3:**
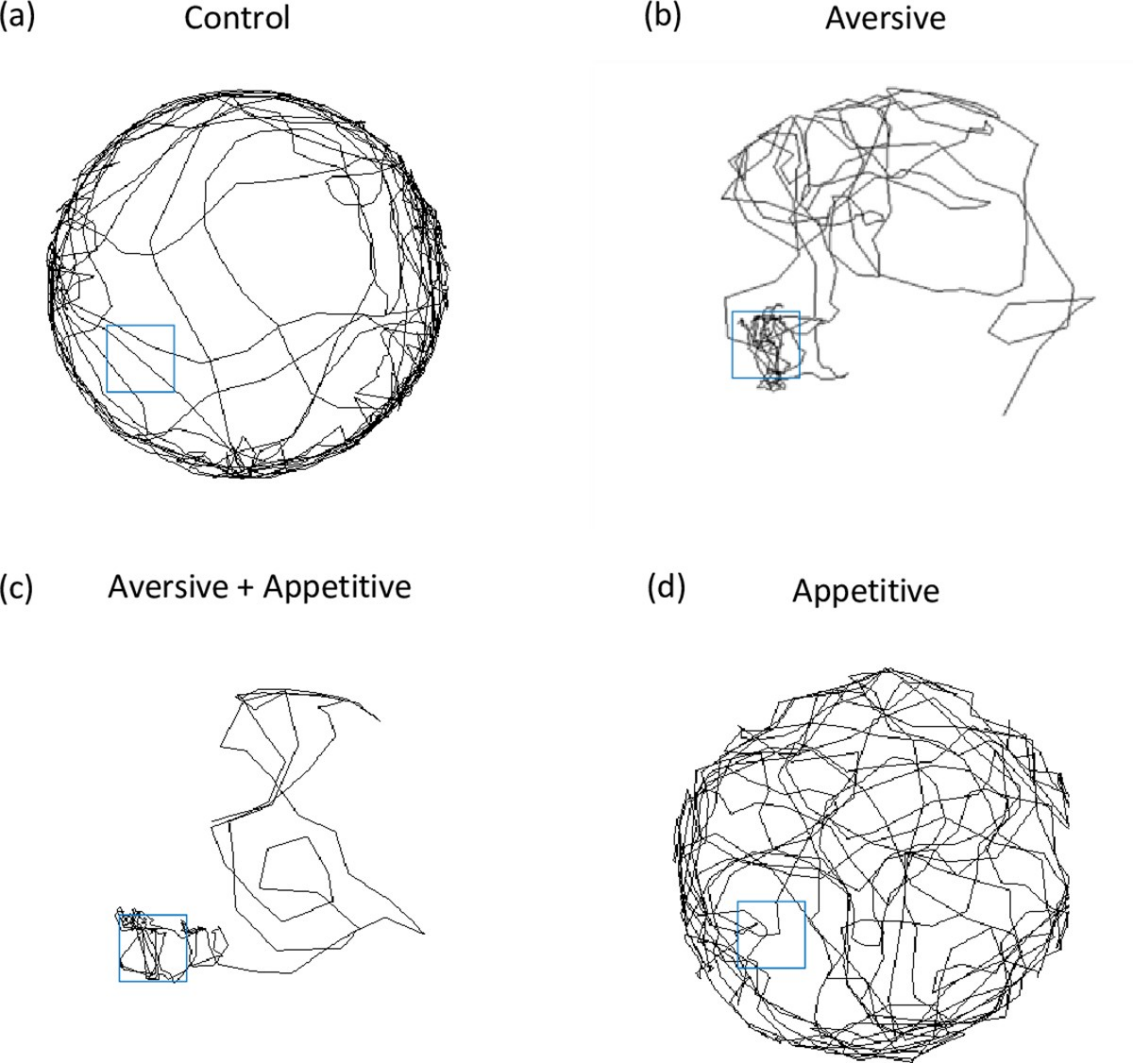
Trajectories of representative bees from the control (a), aversive (b), appetitive (c) and combined aversive and appetitive conditions (d). The blue squares indicate the location of the reward zone (specific to condition) in the arena environment. Bees appear to implement differing exploratory strategies, dependent on the reward or punishment environment they are in. In the control condition (a), individuals often trace concentric paths which delineate the arena boundary. In the aversive condition (b), with a heated floor, individuals were motivated to locate and remain in the cool reward zone. Therefore, trajectories often showed directed exploratory paths out from the reward zone to a facet of the arena. Similar directed trajectories are seen for individuals in the combined aversive and appetitive condition (d). This is not surprising as this is the condition which should provide foragers with the most motivation to remain in the reward zone, with two rewards (sucrose and cool zone) and a punishment in the form of the heated arena floor. Individuals in the appetitive reward environment (c) often tracked more varied paths, not constrained to set routes or areas of the arena.

In all environmental conditions, bees traced complex trajectories (Fig 3 panels a, b, c, d). In each case curvature is seen to occur across a broad range of scales, as evidenced by the presence of nearly straight-line movements with low curvature and the presence of tight turns with high curvature. Across differing environments bees appeared to display varying exploratory trajectories. Individuals tested in the control condition often traced concentric paths, delineating the boundary of the arena (Fig 3). Individuals in the aversive condition located and remained in the cool reward zone for extended periods, making directed exploratory trajectories to a section of the arena’s edge (Fig 3B). Similar trajectories were seen for individuals in the combined aversive and appetitive environment where both a sucrose and cool zone reward were given in the same location (Fig 3C). In the appetitive reward environment individual’s trajectories were more varied, not being constrained to particular routes (Fig 3D).

Individual bees’ trajectories may be governed in part by differing motivations in response to differing training stimuli. When provided with no training stimuli there is no motivation for foragers to complete any task other than escape, resulting in delineating pathways (control group, Fig 3A). Training appears to be most effective in the aversive (Fig 3B) and combined aversive and appetitive (Fig 3C) conditions as foragers are increasingly motivated to take direct paths to and from the reward zone. Nonetheless, these complex, highly unique pathways all have statistical regularities characterised by a simple power law, which holds true irrespective of motivational environment or training regime.

### The speed-curvature relationship

**Fig 4:**
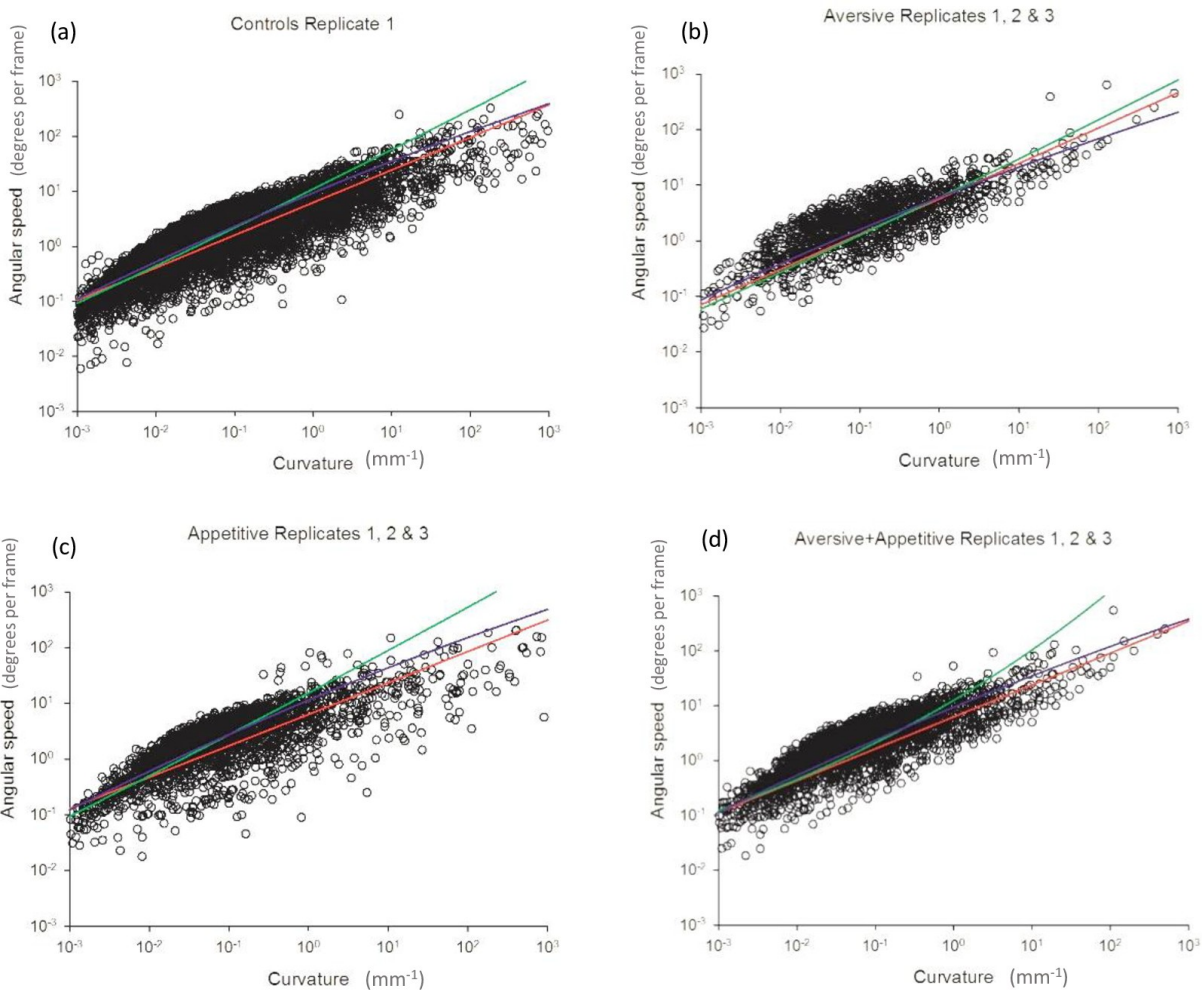
The relationship between angular speed and curvature of path in walking bee trajectories. The two-thirds power law holds true in walking bees across differing environments (control (a), aversive (b), appetitive (c) and combined aversive+ appetitive (d). (a) Scatter plot of instantaneous angular speed plotted against local path curvature at a population level on a log-log scale, for all individuals in the control group. All data points (n = 12224) were sampled at equal time intervals along the trajectories of 14 individual bees. Data was fitted to the power function A(t) = kC(t)2/3 (red line), to stretched exponentials (green line) and log-normal (blue line) functions. Stretched exponentials and log-normals can resemble power-laws and are strongly competing models of the data. (b) Log-log plot of angular speed versus curvature for 7 bees in the aversive group (n = 1081). (c) Log-log plot of angular speed versus curvature for 12 bees in the appetitive group (n = 1835). (d) Log-log plot of angular speed versus curvature for 12 bees in the combined aversive + appetitive group (n = 2309).

A power-law relationship between curvature, C, and angular speed, S, (C = aS^b) will manifest itself as a straight-line (log A = log K + beta log C) on a log-log plot. We tested for such a straight-line relationship by linearly regressing log C on log S for each bee within each environmental condition (Fig 4A, 4B, 4C and 4D). The average (mean) estimates for the power-law exponents are 0.59 (controls, n = 14, range 0.42–0.87), 0.61 (appetitive + aversive, n = 12, range 0.43–0.87), 0.60 (aversive, n = 7, range 0.49–0.94) and 0.57 (appetitive, n = 12, range 0.44–0.8).

The suitability of the power law to describe our data was tested against two competing statistical relationships; stretched exponentials and log-normal like functions (Fig 4A, 4B, 4C and 4D) (see ‘statistical analysis’ methods section for further details). Power laws provide better fits than stretched exponentials, and although good fits are obtained with log-normal functions, they are consistent with the two-thirds power law rule, making the simpler, more elegant power law model the best choice.

### Adherence to a power law across environments

Adherence to the law did not depend on the environment an individual forager was exposed to (see Fig 4A–4D) and the distribution of power exponents did not differ significantly between treatments (including controls) (Kruskal-Wallis ANOVA by ranks, chi-squared = 0.62489, df = 3, p-value = 0.8907 (>0.05)). As would be expected, all treatment exponents were significantly different from zero (Kruskal-Wallis ANOVA by ranks, chi-squared = 32.321, df = 4, p = 1.645e-06**** (<0.00001)).

### Two-thirds or three-quarters?

To determine whether bees’ trajectories adhered more closely to the two-thirds or the three-quarters power law exponent, treatments were tested for significance against populations with assumed power exponents of 0.66 and 0.75.

Treatment populations were highly significantly different from the three-quarters power law exponent (0.75) (Kruskal-Wallis ANOVA by ranks, chi-squared = 17.79, df = 4, p-value = 0.001356** (<0.05)).

However, treatment populations were not found to be significantly different from the two-thirds power law (0.66) (Kruskal-Wallis ANOVA by ranks, chi-squared = 6.0816, df = 4, p-value = 0.1931 (>0.05)). However, Fig 2 shows that although treatment groups did not differ significantly from 0.66, the medians of treatment groups vary around a 0.55 power exponent line. Populations were found to not significantly differ from this 0.55 power exponent either (Kruskal-Wallis ANOVA by ranks, chi-squared = 1.7447, df = 4, p-value = 0.7826 (>0.7826).

## 4. Discussion

Locomotive patterns are frequently complex but do, nonetheless, have surprising regularities (primitives) that may provide insights into the underlying generative mechanisms for movement and into motor planning. These regularities take the form of power-laws that have been shown to characterise not only curvature[1], but also the duration of movement bouts and pauses[26].

Our work in *Bombus terrestris* supports previous findings in *Drosophila* larvae[5] that the power laws which govern voluntary human behaviours[1,3,4,6] also govern the behaviours of less complex organisms. Remarkably, this law holds, not just across vastly different locomotive methods and speeds (walking[6], drawing[1], crawling[5]), but also across greatly differing organisms (human[1,3,4,6] and non-human primates[8], Diptera[5], and now Hymenoptera).

The explanations for these power laws within movement patterns are contentious with contrasting hypotheses for their existence. Originally ascribed to central motion planning by the nervous system[8,9] it was thought that the existence of the relationship between speed and curvature could not be a result of muscular properties and limb dynamics[10]. This is supported by the observation that the law holds true for human drawing under isometric conditions[27]. Notably, the speed-curvature power law is also corroborated across widely diverse taxa. Evidence that the law originates as a result of decoding complex cortical processes is apparent in the motor cortical control of Rhesus monkey hand movements, as population vectors in the motor cortex obey the power law during drawing[8], adding weight to the central planning origin hypothesis.

*Drosophila* Larval locomotion power exponents have been recorded to deviate from the two-thirds exponent reported for human voluntary movements[1,3,4,6], at closer to three-quarters[5]. The researchers suggest that these findings prove a role for dynamic effects adding on purely kinematic constraints[5]. In support of this notion, the power exponent recorded for human drawing shifts closer to this value of three-quarters (0.73) when drawing underwater[28], suggesting that power laws can indeed be governed by kinematic constraints. Our analyses suggest that, in walking bumblebees, a power law exponent between 0.55 and 0.66 (two-thirds) better defines movements than the near 0.75 exponents previously reported for *Drosophila*[5] and constrained human movements[28]. Our evidence further supports the idea that exponents are forced closer to the three-quarters value when kinematic constraints are present, as our constraint-free bees have a generally much lower exponent at closer to two thirds.

However, other studies take a less definitive approach, suggesting that biomechanical factors and central planning may interact to constrain kinematic movement aspects, limiting the degrees of freedom which they can take[29]. An extension of this, the minimum jerk hypothesis[3,9] states that the law exists to maximize smoothness, selecting for jerk-free, stable, controllable movements. The occurrence of these laws across organisms could be seen to support a convergent evolution theory of a jerk-free movement mode which remains behaviourally efficient across organisms of different size, complexity, and phyla. Maximally smooth movements may seem to be without biological significance for grounded invertebrates, like crawling *Drosophila* larvae[5] and walking bumblebees. However, they could, nonetheless, be adaptive for airborne invertebrates, allowing for downwind flights in the absence of visual cues for orientation. Such common orientation has been widely documented since the advent of entomological radar, and allows noctuid fliers to add their flight speed to the wind speed, so maximizing their dispersal[30]. Our analysis suggests that this ability is a spandrel that predates flight, lying dormant in terrestrial movements.

Contrarily, the pervasiveness of the law may be an inconsequential by-product of the noise inherent to central pattern generators (CPGs)[31]. Or more positively, an accidentally advantageous property of noise, as somewhat paradoxically, noise may result in maximally smooth, controllable movement. Possibly, the law may stem from simple harmonic motions[32], such as those outputted by CPGs when combined with muscular viscoelastic properties[2]. However, this hypothesis seems unrealistic when considering the power law in walking bees as we report here.

Our findings, together with those of Gomez-Marin et al.[5] for *Drosophila* larvae, are suggestive of common mechanics of model switching in the locomotion of limbless and legged animals. As first suggested by Kuroda et al.[33] who noted similarities between leg-density waves of centipedes and millipedes and the locomotive waves of limbless animals. Our findings hint at a deeper analogy. Marken & Shaffer[34] have argued that these power laws are artefacts of the calculations themselves. However, this seems improbable, as the law is shown to persist regardless of its calculation methodology[35].

Any tendency to walk around the perimeter of the circular arena (of radius r = 10 cm) either in part or wholly will be associated with a curvature of radius R = r. Our data for this curvature is consistent with the overall power-law scaling seen across all radii and is not anomalous. This suggests that the circular geometry of the arena is not impacting on the speed-curvature power law. This may not be true of other geometries, such as squares, who’s corners might be associated with high curvatures.

In our analyses, individual bee’s tracking data were pooled within each learning environment. This allowed us to collectively compare each training group to differing statistical models and to examine a potential training environment impact on power law exponents. We acknowledge that this approach minimises the role of intra-individual behavioural variation often seen in bees[36]. Although we have not examined it here, future studies could examine the impact of this intra-individual variation on power law exponents between bees and across learning experience.

The multitude of evidence for varying originating mechanisms suggests that the origins of such power laws are most likely pluralistic in nature and potentially constraints vary across organisms. Nonetheless, the pervasiveness of these multiple scaling laws, across both taxa and locomotive mode, could imply an underlying driver. The notion that scale-free movements are intrinsic[11] suggests universal scaling laws could present an optimal behavioural template which may then be favoured by natural selection.

Nonetheless, this might be overemphasizing the role of evolution as the fundamental determinate of behaviour, and underemphasizing the role of physical laws and mechanical limitations, as exemplified by the minimum jerk hypothesis[3,9]. As animals, may simply be predisposed to have jerk-free movements due to physical constraints. The argument for process structuralism[37], in which mathematical laws supersede natural selection as a “shaping agency”[38] may therefore be more applicable. This resonates with the occurrence of Levy walks; movement patterns that are characterised by power-laws and seen across taxa from single cells to humans. In many cases these appear to be shaped by physical constraints rather than by natural selection[39].

Understanding the basal behavioural templates behind organisms’ locomotive trajectories may provide a tool for behavioural study. Biological stressors, such as disease, have been shown to cause deviations from this optimal behavioural template[40]. Power laws may therefore provide a diagnostic tool for the sub-lethal impact of such stressors at a finer scale.

Our work with *Bombus terrestris* is one of the few examples of the speed curvature power law outside human movements. Supporting the notion of an optimal behavioural template which is pervasive across movement modes and organisms as a result of kinematic constraints. The discovery of this null template in *Bombus terrestris* may add a tool to the arsenal of scientists, allowing us to better study potential sublethal disruptors of optimal behaviour.

## Supporting information

S1 Dataset Raw centroid tracking data: This data was used to calculate speed-curvature power laws from bee trajectories. (ZIPX)

S1 Appendix Data filtering and pre-processing: Additional information is provided on the processing and filtering of the raw centroid tracking data prior to analyses. (DOCX)
