# Supplementary material for: Do bumblebees have signatures? Demonstrating the existence of a speed-curvature power law in Bombus terrestris locomotion patterns
Source: PLoS One. 2020 Jan 15;15(1):e0226393. doi: 10.1371/journal.pone.0226393 (PMC6961848; doi:10.1371/journal.pone.0226393)
Supplement: S1 Appendix — (DOCX) [file pone.0226393.s002.docx]

**Data filtering and pre-processing:**

For the data analysis, the x, y coordinates and corresponding timestamps for whole trajectories from the centroid tracking were used to compute angular speed A(t) and curvature C(t) using standard differential geometry. Positional data were not filtered to reduce any noise or cusps that may be present before computing speed and curvature. To assess the impact of filtering we compared analyses based on the raw, consecutive positional fixes (x_1_,y_1_), (x_2_,y_2_), (x_3_,y_4_) with those based on filtered positional fixes ½ (x_1_+x_2_,y_1_+y_2_), ½(x_3_+x_4_, y_3_+y_4_),…, obtained by averaging over pairs of consecutive positional fixes. Such filtering did not affect the outcomes of the analysis. This is illustrated in Fig A. The effects of this filtering on our trajectory data are shown in Fig B. To further assess the impact of any noise and cusps we carried out conditional analyses on speed-curvature data associated exclusively with relatively low velocities and low accelerations. We found that such conditioning does not impact significantly on our estimates for the power-law exponents characterizing the speed-curvature power-law relationship. This is illustrated in Fig C. The results suggest that the high inter-quartile range of the β fitting shown in Fig 4 are not related to the absence of any filtering or interpolation before the differentiation of positional data used to compute angular speed and curvature.


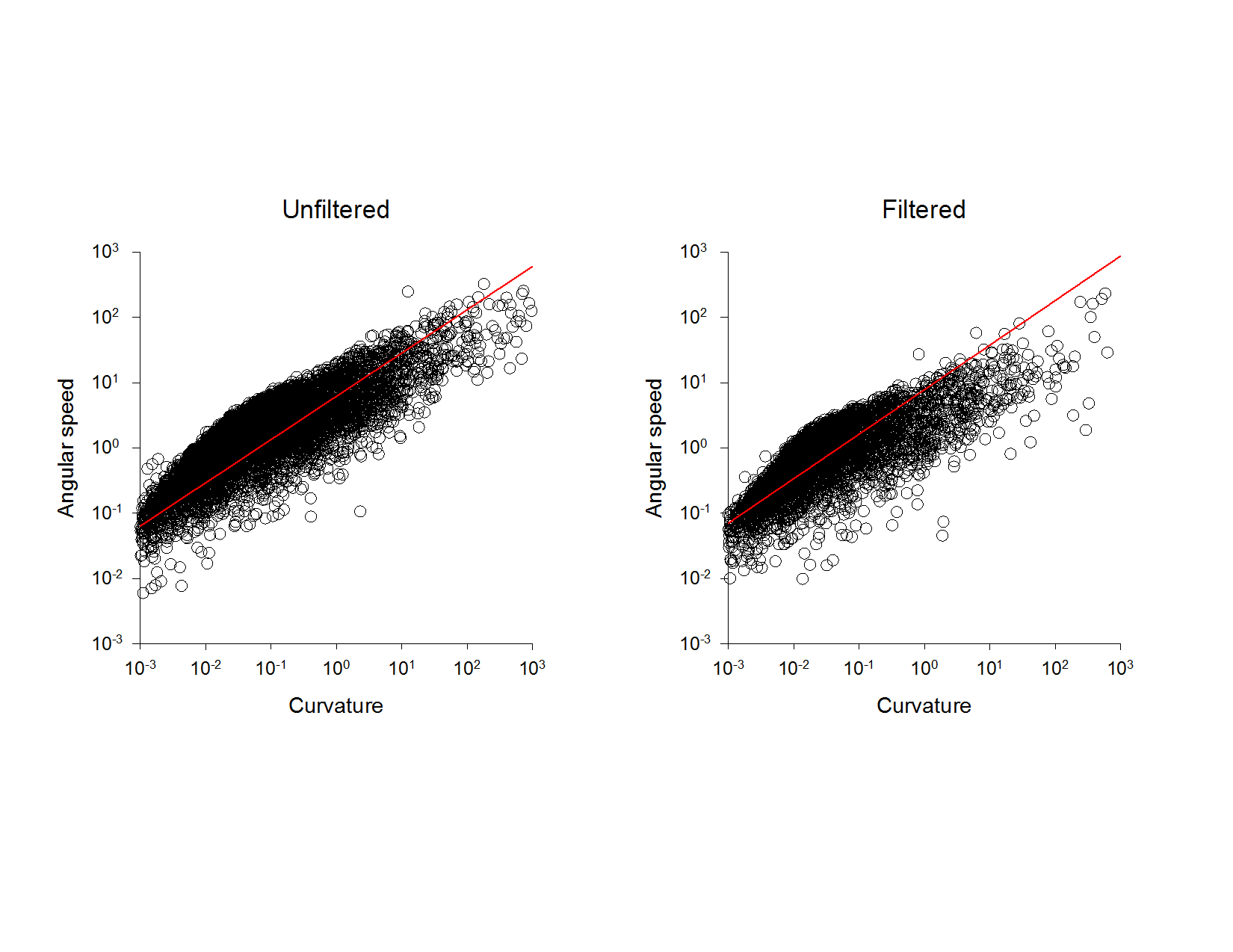


**Fig A.** This figure demonstrates that filtering implemented prior to data analyses does not affect the outcome of the analyses. Analyses are presented here for the Control Replicate 1 dataset with and without filtering (smoothing). In both cases the best fit power-law exponent is 0.68.


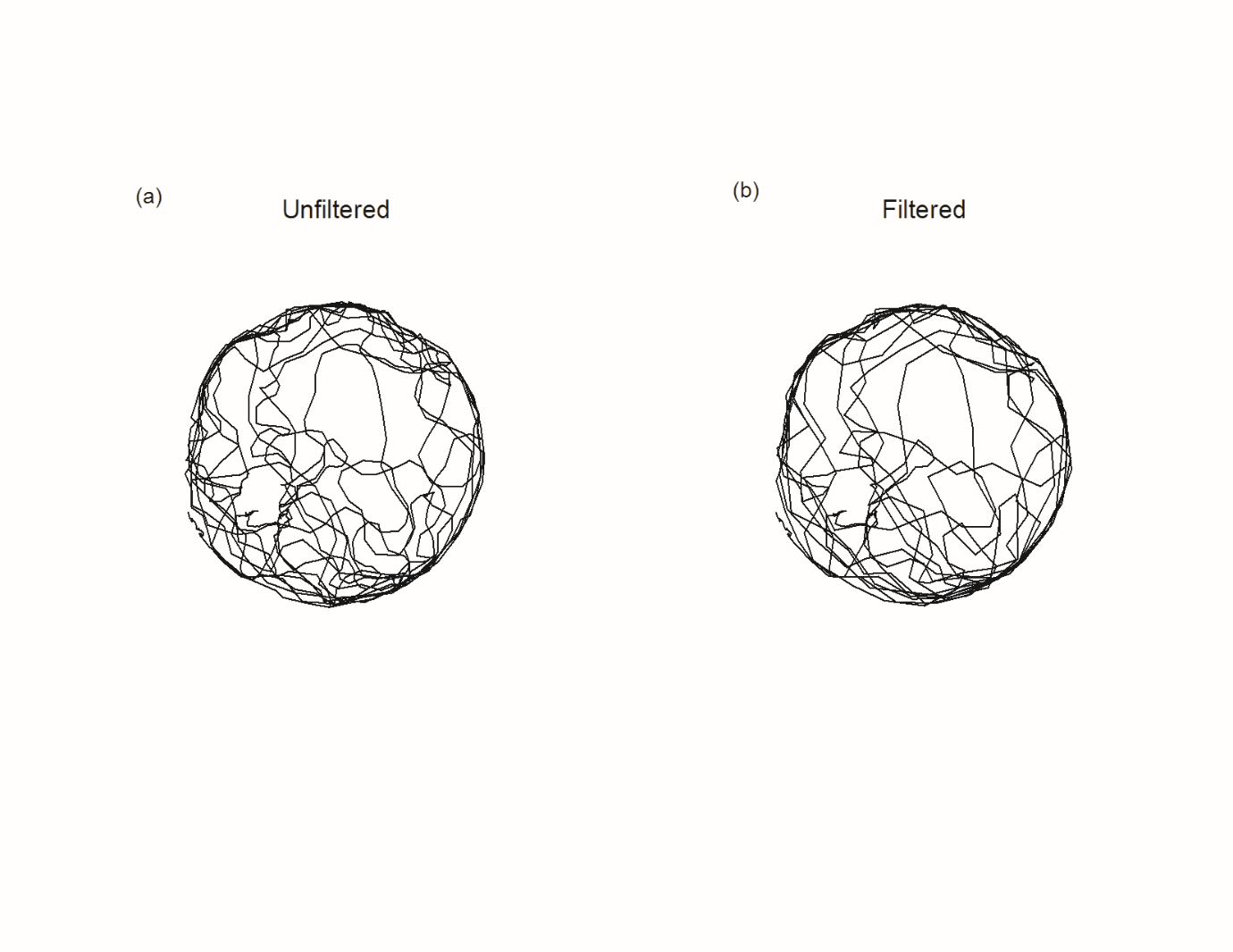


**Fig B.** This figure demonstrates the effect of filtering on our trajectory data. (a) Demonstrates an example of a single bee’s unfiltered trajectory. (b) Demonstrates the same bee’s post-filtered trajectory.

*
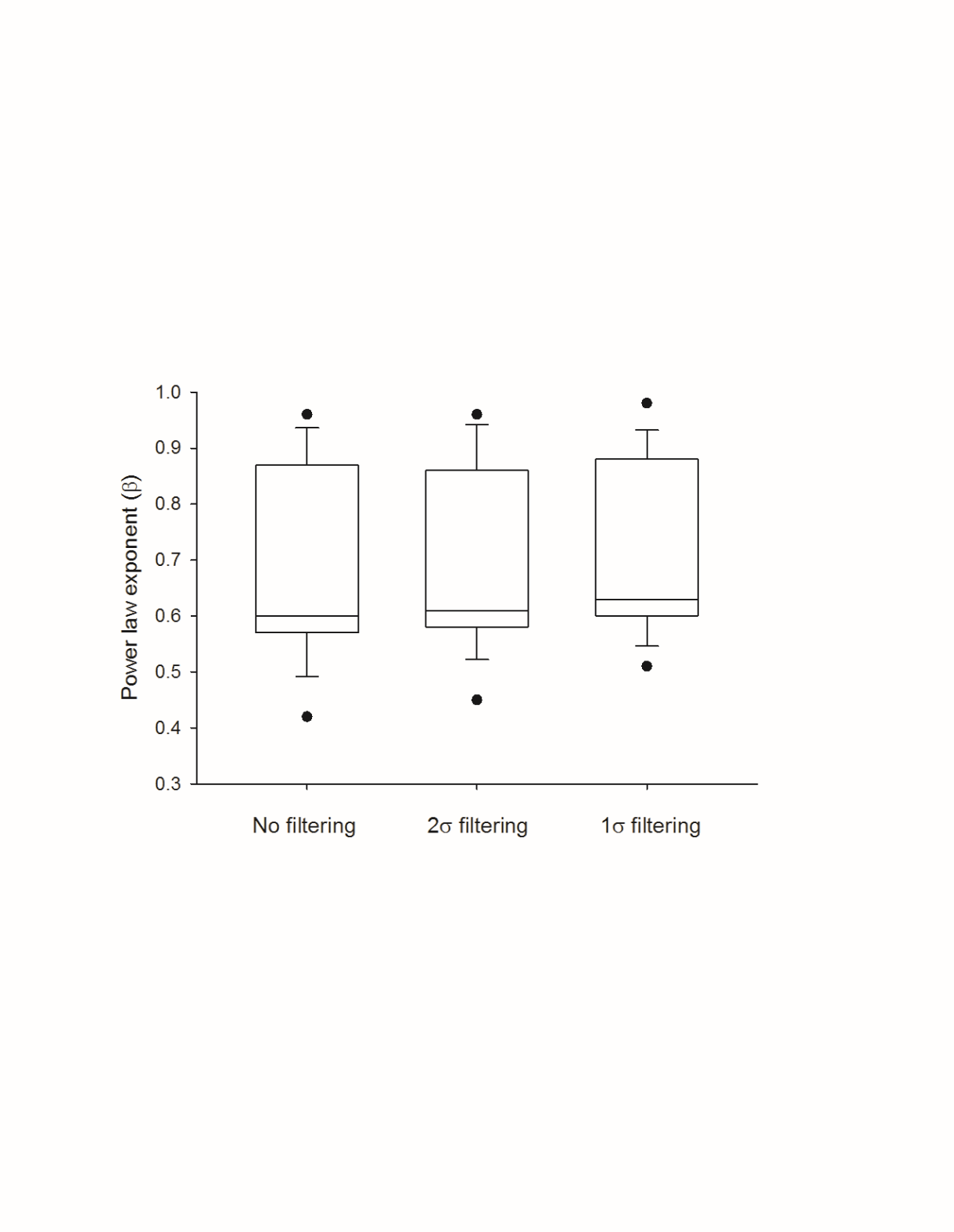
*

**Fig C.** This figure demonstrates that our estimates for the power-law exponents, *β*, are not unduly influenced by the presence of noise and cusps, that are expected to be associated with high-velocities and/or high-accelerations. Results are shown for Replicate 1 Controls (15 individuals): full dataset with no filtering (left); a reduced dataset excluding speeds and accelerations (middle) and; a reduced dataset excluding speeds $v>\left\langle v^{2}\right\rangle^{1/2}$ and accelerations $a>\left\langle a^{2}\right\rangle^{1/2}$(right). The conditioning removes around 10% and 50% of the data from the dataset.
